# Oral microbiome contributes to the failure of orthodontic temporary anchorage devices (TADs)

**DOI:** 10.1186/s12903-023-02715-7

**Published:** 2023-01-17

**Authors:** Ningrui Zhao, Qian Zhang, Yanning Guo, Shengjie Cui, Yajing Tian, Yidan Zhang, Yanheng Zhou, Xuedong Wang

**Affiliations:** 1 Department of Orthodontics, Peking University School and Hospital of Stomatology & National Center of Stomatology & National Clinical Research Center for Oral Diseases & National Engineering Laboratory for Digital and Material Technology of Stomatology & Beijing Key Laboratory of Digital Stomatology & Research Center of Engineering and Technology for Computerized Dentistry Ministry of Health & NMPA Key Laboratory for Dental Materials, No. 22 Zhongguancun South Avenue, Beijing, 100081 China; 2 Central Laboratory, Peking University School and Hospital of Stomatology, 22# Zhongguancun South Avenue, Haidian District, Beijing, 100081 China

**Keywords:** Microbiology, Orthodontics, Temporary anchorage devices (TADs), Next-generation sequencing

## Abstract

**Background:**

The stability of temporary anchorage devices (TADs) is critical in orthodontic clinics. The failure of TADs is multifactorial, and the role of the oral microbiome has not been clearly defined. Herein, we attempted to analyze the contribution of the oral microbiome to the failure of TADs.

**Methods:**

Next-generation sequencing was adopted for analyzing the microbiome on the TADs from orthodontic patients. 29 TADs (15 failed TADs and 14 successful TADs) were used for 16S rRNA gene sequencing. A total of 135 TADs (62 failed TADs and 73 successful TADs) were collected to conduct metagenomic sequencing. Additionally, 34 verified samples (18 failed TADs and 16 successful TADs) were collected for quantitative real-time polymerase chain reaction analysis (qRT-PCR).

**Results:**

Successful and failed TADs demonstrated discrepancies in microbiome structure, composition, and function. Clear separations were found in β-diversity in 16S rRNA gene sequencing as well as metagenomic sequencing (*p* < *0.05*). Metagenomic sequencing showed that *Prevotella intermedia, Eikenella corrodens, Parvimonas spp., Neisseria elongata,* and *Catonella morbi* were enriched in the failed groups. qRT-PCR also demonstrated that the absolute bacteria load of *Prevotella intermedia* was higher in failed TADs (*p* < *0.05*). Considering functional aspects, the failed group showed enriched genes involved in flagellar assembly, bacterial chemotaxis, and oxidative phosphorylation.

**Conclusions:**

This study illustrated the compositional and functional differences of microorganisms found on successful and failed TADs, indicating that controlling bacterial adhesion on the surface of TADs is essential for their success rate.

**Supplementary Information:**

The online version contains supplementary material available at 10.1186/s12903-023-02715-7.

## Background

Anchorage control is of great importance for orthodontists. Temporary anchorage devices (TADs) are flexible and easy-to-operate devices implanted in the alveolar bone that provide persistent orthodontic forces and orthodontic anchorage. They have been extensively used in orthodontic clinics, and they are a reliable skeletal anchorage for anterior teeth retraction, molar protraction, space closure, molar distalization, and supra-erupted teeth intrusion [1, 2].

The stability of TADs is the premise for their strong anchorage function. However, mobility and failure of TADs are much higher than in dental implants. The failure rate of TADs is 10%-20% [3, 4], posing a great challenge to orthodontists, and the elevated level of failure reduces orthodontic efficacy. Factors that contribute to TAD failure are complicated and ambiguous. Several factors have been reported to be related to TAD failure, including site-related factors, implant-related factors, TAD design, loading force application, and inflammation around the TAD [5–9].

The oral cavity is a unique microenvironment, which harbors distinct microbial communities. The deleterious shift of the microbiota balance is generally recognized as an oral disease driver [10]. In the disease model of periodontitis and peri-implantitis, microbiomes could induce host inflammatory responses and eventually cause bone resorption in surrounding areas. However, TADs differ from the dental implant in the insertion site as well as the non-osseointegration healing process. Therefore, the evidence of microorganisms' negative effects on dental implants might not be applied to TADs. In the case of TADs, it has been suggested that microbiota dysbiosis could hamper the healing process after the insertion of TADs. The insertion process itself triggers host inflammatory responses. If bacterial invasion occurs during the healing process, loss of stability might happen [11]. In order to verify this hypothesis, previous studies have attempted to detect pathogenic bacteria around failed TADs with polymerase chain reaction (PCR) and checkerboard DNA-DNA hybridization technique but failed to build up the connection [12–14]. Previous work has only focused on the abundance and detection rate of single bacteria, however, the phylogenetic and functional composition changes of microbiota around failed TADs remain obscure.

With the rapid development of sequencing technology, new strategies have been implemented for unveiling the relationship between the microbiome and oral health. Next-generation sequencing (NGS) enables us to gain overall perspectives of oral diseases, including periodontitis, peri-implantitis, dental caries, apical abscesses, and oral cancers [15–17]. In this study, we use 16S rRNA sequencing, which could reflect the overall picture of the microbiome on TADs. Metagenomic sequencing was also carried out to provide specific insights into phylogenetic assessments and functional analysis.

Therefore, we hypothesized that there were different microbiota colonized around TADs under different stable conditions. The objective of our study was to reveal the differences in the structure, composition, and function of microbiome colonized around TADs between failed and successful TADs with amplicon sequencing and metagenomic sequencing.

## Methods

### Patients recruitment and sample selection

All participants were orthodontic patients under treatment at the Peking University School and Hospital of Stomatology. TADs were applied as anchorage reinforcement during their treatment. Informed consent forms were signed by all participants enrolled in the study. The Ethics Committee of the Peking University School and Hospital of Stomatology approved the study under PKUSSIRB-202060204. TADs were grouped as failed and successful TADs. Failed TADs were defined as TADs showing severe mobility with signs of inflammation and were unable to serve as anchorage devices before the end of the treatment. Successful TADs conversely maintained stability until the end of the treatment. No signs of inflammation or infection were observed.

All participants were selected under the following conditions: (1) 12–45-year-old individuals; (2) received periodontal examination and were suitable for orthodontic treatment; (3) non-smokers and did not consume alcohol; (4) physically healthy with no concomitant disease; (5) not pregnant; (6) no antibiotic intake three months prior to treatment.

Self-drilling TADs were implanted by several experienced orthodontists using the same surgical technique. The titanium TADs were 7 mm or 8 mm in length, 1.5 mm in diameter, and manufactured by Zhongbang Medical Treatment Appliance in Xi’an, China. TADs attained primary stability right after implantation. There was no tooth root damage observed. All participants were asked to brush the TAD twice a day. The TADs were all loaded one month after implantation. The “Micropower” package (http://github.com/brendankelly/micropower) was used to assess the sample size. Demographic parameters including age, sex, oral hygiene condition, periodontal condition, and Angle’s classification were recorded.

### DNA extraction

After removal, TADs were immersed in 500 μL normal saline solution in the nonpyrogenic microcentrifuge tubes. The solutions were collected and stored at − 80 ℃ for further analysis. The test tubes were sonicated for 20 min in an ultrasonic machine (SB-3200DTN, Ningbo, China) before extraction. The microcentrifuge tubes were later centrifuged for 15 min at 8,000 rpm. The pellet was then used for DNA extraction.

The pellet was incubated for 30 min at 37 °C after adding 180μ L lysozyme (Solarbio, Beijing, China). Genomic DNA was extracted using a QIAamp DNA Mini Kit (QIAGEN, Hilden, Germany) according to the manufacturer’s instructions. The purity and integrity of DNA were examined using a Nanodrop 8000 spectrophotometer (Thermo Fisher Scientific, Carlsbad, California) and by 1% agarose gel electrophoresis.

### 16S rRNA gene sequencing and analysis

Amplification of V3-V4 region of the bacterial 16S ribosomal DNA was carried out by PCR using primers 341F (5'-CCTACGGGRSGCAGCAG-3') and 806R (5'-GGACTACVVGGGTATCTAATC-3') in a total reaction volume of 30 μL (15 μL KAPA Library Amplification ReadyMix, 1 μL primer, 50 ng template DNA, and ddH2O to volume). Thermocycling conditions included an initial denaturation step at 95 °C for 3 min, then 30 cycles of 20 s at 98 °C, 15 s at 58 °C, and 20 s at 72 °C, with a final 5-s extension at 72 °C. A 2% agarose gel was used to separate PCR products and an AxyPrep DNA Gel Extraction Kit (Axygen Biosciences, Union City, CA, USA) was used to extract amplicons. Invitrogen's Qubit 2.0 library was used to quantify the library. Sequencing was performed on an Illumina MiSeq platform (Illumina, San Diego, CA, USA) according to standard procedures (2 × 250 bp paired-end) after amplicons were pooled to equalize concentrations.

Vsearch (version 2.15) was used to merge raw paired-end sequences based on their overlapped tags. A maximum of five mismatches was allowed. After demultiplexing, Vsearch was then able to obtain clean reads by removing the barcode and primers, with an error rate no higher than 1%. Unoise3 in USEARCH (version.10) denoised the data into amplicon sequence variance (ASV). Vsearch then created the feature table and detected and removed the chimeras. We referred to the RDP Classifier [18] and the Human Oral Microbiome Database (HOMD) [19] as database sources.

The downstream analysis was started by randomly rarefying the pre-processing sequences to combat the effects of the variable sequencing depths. We adopted Usearch to calculate α-diversity indices and β-diversity indices. R package ‘Amplicon’ (https://github.com/microbiota/amplicon) was used to perform principal coordinate analysis (PCoA). ASVs were further categorized into microbial taxa (kingdom, phylum, class, order, family, and genus).

### Metagenomic sequencing and analysis

Illumina MiSeq platform (Illumina, San Diego, CA, USA) was used for the sequencing and PE150 strategies were used. DNA libraries were constructed by inserting approximately 500 bp per sample under the guidance of the Illumina TruSeq DNA Sample Prep v2 Guide (Illumina, Inc., San Diego, CA, USA). Agilent 2100 bioanalyzer (Agilent Technologies,Wokingham, UK) and the Agilent 2100 DNA 1000 kit were used to assess the quality of the library. The sequence depth of each sample must be at least 5 Gbp. Illumina raw reads were screened based on the presence of the following conditions: (1) adaptor contamination; (2) low quality (Q < 20) bases; (3) more than three ambiguous N bases; (4) high-quality bases (Phred score ≥ 20) < 60%. After screening, SOAPaligner (version 2.21) was used to align clean reads to bacterial genome sequences deposited in the National Center for Biotechnology Information GenBank. Reads that aligned to the host genome were abated.

SOAPaligner 2.21 was used to detect bacteria, viruses, fungi, and archaea in the NCBI database (https://ncbi.nlm.nih.gov/). The aligned reads were then further classified into microbial taxa for downstream analysis. Species identification was also performed using Kraken2 (ver.2) with the miniKraken database (https://ccb.jhu.edu/software/kraken2/). The number of species that were detected in each sample was counted. α-diversity indices and β-diversity indices were calculated after rarefication according to Liu et al.[20]*.*

SOAPdenovo (Version 1.05) was used to preprocess reads, which were then assembled for each sample using a series of k-mers (51, 55, 59, 63). Assembled scaffolds were then divided into contigs at ambiguous Ns. Contigs with a minimum size of 500 bp were retained for analysis, and N50 k-mers were selected for final assembly. MetaGeneMark software (http://exon.gatech.edu/GeneMark/metagenome/Prediction/) was used to predict open reading frames (ORFs) in the assembled scaffolds, and ORFs with less than 100 bp were trimmed. CD-HIT (ver 4.5.7) was used to determine the non-redundant gene catalog based on a pair-wise comparison of predicted ORFs (gene length > 100 bp). Redundant sequences were considered when the two sequences with coverage ≥ 90% and identity ≥ 95%. The longer one was regarded as the representative. The final non-redundant gene catalog contained 1,704,942 ORFs with an average length of 570.8 bp.

By using SOAPaligner, the reads were aligned to the genes in the nonredundant catalog. The calculation of gene abundance was carried out according to Qin et al. [21]. Annotations were made to genes using BLAST (Version 2.2.28 +) against the KEGG (Kyoto Encyclopedia of Genes and Genomes) database (https://www.genome.jp/kegg/brite.html). We accumulated the relative abundance of all orthologous genes to produce the relative abundance of each KEGG ortholog.

### Statistical analysis

Student's t-test was implemented to determine age variations between groups. Fisher’s exact test and Pearson’s chi-squared test were performed to evaluate sex variations, oral hygiene conditions, periodontal conditions, and Angle’s classification. We have conducted the MaAsLin (Multivariate Analysis by Linear Models) (http://huttenhower.sph.harvard.edu/galaxy/) to address potential biases. Similarly, Wilcoxon’s test was used to compare TAD retention days in the oral cavity. Differences in α-diversity indices were compared using Student's t-test. β-diversity variations were measured by pairwise permutational multivariate analysis variance (PERMANOVA) through the “Vegan” package (https://cran.r-project.org/web/packages/vegan/index.html). The Receiver Operating Characteristic curve (ROC curve) was generated by SPSS (ver.26) and was visualized by Graphpad (ver.9.0). Wilcoxon’s test was used to determine differential species and functions between groups. Correlation between bacteria was measured with Spearman’s correlation coefficients. We employed Cytoscape (ver. 3.5.1) to demonstrate the inter-species correlations through the network. P < 0.05 was considered statistically significant.

### Quantitive real-time PCR (qRT-PCR) analysis

In order to quantify the bacterial load, universal primers and specific primers targeting *Prevotella intermedia* were selected [22]. The universal primers were 8F-AGAGTTTGATCMTGGCTCAG and 361R-CYIACTGCTGCCTCCCGTAG [23]. Specific primers for *Prevotella intermedia* were F-CGTACGGGAGTGTTACTGACG and R-CTTTCGCTTAGCCGCTAACG [24]. The amount of DNA in the samples was adjusted to 10 ng/μL. Each qRT-PCR was performed with 2 biological replicates. The qRT-PCR reactions were performed in 20 μL reaction volumes and contained 10 μl Fast Power SYBR green PCR Master Mix, 1 μM of each primer pair, 1 μl DNA template and ddH2O to volume. The thermocycler conditions included a denaturation step for 2 min at 95 °C, then 40 cycles of 15 s at 95 °C and 1 min at 60 °C. The DNA of *Porphyromonas gingivalis* was used as a standard for total bacteria*.* The standard bacteria of *Prevotella intermedia* and *Porphyromonas gingivalis* were 10,000 pg/μL, 1,000 pg/μL, 100 pg/μL, and 10 pg/μL, and these concentrations were used to produce a standard curve. The mean value was used for analysis. Wilcoxon’s test was used to determine the significance of differences.

## Results

### Sample information

**Fig. 1:**
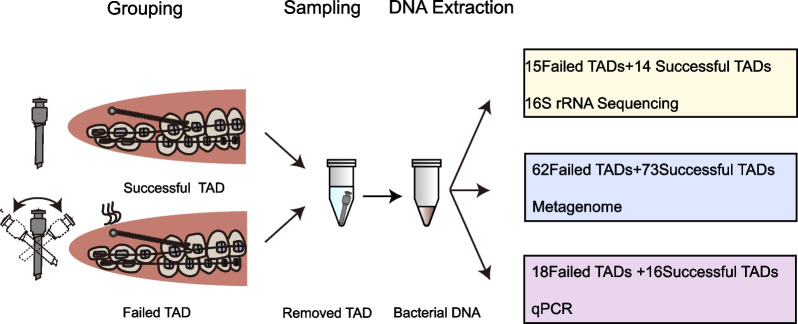
The flow chart of this study shows the workflow of this microbiota analysis

We collected 29 TADs (15 failed TADs from 15 patients and 14 successful TADs from 14 patients) for 16S rRNA gene sequencing. For the metagenomic analysis, 135 TADs were collected (62 failed TADs from 47 patients and 73 successful TADs from 40 patients). In order to meet the DNA quantity requirement of metagenomic sequencing, we merged 10–12 TADs into one sample, producing six samples in the failed group and six samples in the successful group. Another 34 verified TADs (18 failed TADs from 14 patients and 16 successful TADs from 15 patients) were collected for qRT-PCR. Demographic parameters for 16S rRNA gene sequencing, metagenomic analysis, and qRT-PCR analysis are listed in Tables 1, 2, and 3. No difference was observed in age, sex, oral hygiene conditions, periodontal conditions, and Angle’s classification between groups. No detection between microbial measurements and clinical metadata (age, oral hygiene condition, periodontal condition, and Angle’s classification) was found in the MaAsLin analysis. The TAD retention time in the oral cavity was shorter in the failed group (*p* < *0.001*). The design and flow path of our experiments are shown in Fig. 1.

**Table 1:** Demographic and clinical features of subjects under 16S rRNA sequencing

Variable	Successful (n = 14)	Failed (n = 15)	*P*
Age (y)	27.50 ± 7.66	25.13 ± 5.40	0.342
Sex ratio (male: female)	3:11	0:15	0.1
Time in oral cavity (d)	701.29 ± 320.65	161.80 ± 97.32	< 0.001*
Oral hygiene condition			0.068
Good	4/14	2/15	
Fair	7/14	13/15	
Poor	3/14	0/15	
Periodontal condition			0.710
Healthy	0/14	0/15	
Gingivitis	6/14	5/15	
Periodontitis	8/14	10/15	
Angle’s classification			0.857
I	2/14	4/15	
II	10/14	9/15	
III	2/14	2/15	

*Significant difference between successful and failed TADs

**Table 2:** Demographic and clinical features of subjects under metagenomic sequencing

Variable	Successful (n = 73)	Failed (n = 62)	*P*
Age (y)	26.63 ± 7.26	26.21 ± 7.65	0.744
Sex ratio (male: female)	10:63	10:52	0.692
Time in oral cavity (d)	787.15 ± 309.51	134.46 ± 176.54	< 0.001*
Oral hygiene condition			0.058
Good	6/73	14/62	
Fair	54/73	37/62	
Poor	13/73	11/62	
Periodontal condition			1.00
Healthy	10/73	9/62	
Gingivitis	23/73	19/62	
Periodontitis	40/73	34/62	
Angle’s classification			0.288
I	25/73	14/62	
II	40/73	42/62	
III	8/73	6/62	

*Significant difference between successful and failed TADs

**Table 3:** Demographic and clinical features of subjects under qRT-PCR

Variable	Successful (n = 16)	Failed (n = 18)	*P*
Age (y)	30.56 ± 7.81	25.16 ± 7.87	0.054
Sex ratio (Male: Female)	1:15	2:16	0.55
Time in oral cavity (d)	717.31 ± 321.51	219.5 ± 219.16	< 0.001*
Oral hygiene condition			0.113
Good	4/16	3/18	
Fair	9/16	15/18	
Poor	3/16	0/18	
Periodontal condition			0.778
Healthy	1/16	3/18	
Gingivitis	5/16	4/18	
Periodontitis	10/16	11/18	
Angle’s classification			0.845
I	5/16	4/18	
II	10/16	13/18	
III	1/16	1/18	

*Significant difference between successful and failed TADs

### Diversity of successful and failed TADs

When processing 16S rRNA gene sequencing, 840,667 clean reads were obtained. The sequences were 28,988 on average, which eventually clustered to 1,084 ASVs.

**Fig. 2:**
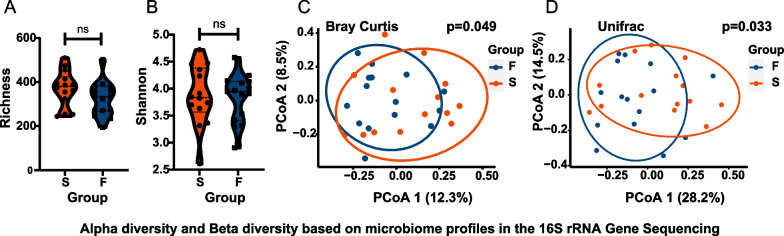
α-diversity and β-diversity based on microbiome profiles from 16S rRNA Sequencing. **A** A boxplot of α-diversity richness between groups (*p* = *0.126*). **B** A boxplot of α-diversity Shannon index between groups (*p* = *0.819*). **C** Principal coordinate analysis (PCoA) based on Bray Curtis distances is shown for the failed groups (blue) and successful group (red) (p = 0.049). **D** Principal coordinate analysis (PCoA) based on weighted UniFrac distances (*p* = *0.033*)

We first evaluated α-diversity and β-diversity to reflect overall differences (Fig. 2). Failed TADs presented lower α-diversity in Richness and Shannon indexes. This difference, however, was not significant compared with successful TADs (*p* = *0.126, p* = *0.819*) (Fig. 2A, B). β-diversity revealed significant differences between failed TADs and successful TADs. Bray Curtis distances as well as weighted Unifrac distances demonstrated the two groups in separate clusters (*p* = *0.049, p* = *0.033*) (Fig. 2C, D).

**Fig. 3:**
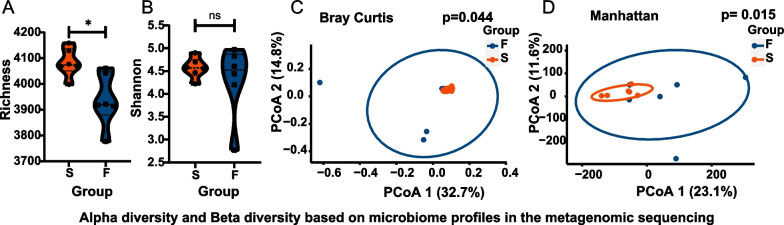
α-diversity and β-diversity based on microbiome profiles in the metagenomic sequencing. **A** A boxplot of α-diversity richness between groups (*p* = *0.013*). **B** A boxplot of α-diversity Shannon index between groups (*p* = *0.446*). **C** Principal coordinate analysis (PCoA) based on Bray Curtis distances (*p* = *0.044*). D. Principal coordinate analysis (PCoA) based on weighted UniFrac distances (*p* = *0.015*)

In the metagenomic analysis, a total of 353,606,794 clean reads were obtained. The sequences were 29,467,233 on average in the 12 samples. Failed TADs demonstrated significantly lower α-diversity by the Richness index (*p* = *0.013*) (Fig. 3A). No difference was observed in the Shannon index (*p* = *0.446*) (Fig. 3B). β-diversity based on Bray Curtis and Manhattan distances showed different group clusters (*p* = *0.044, p* = *0.015*) (Fig. 3C, D). The failed TADs demonstrated greater inter-group discrepancies.

In the amplicon sequencing, microbiome composition was analyzed at the phyla and genera levels. At the phylum level, *Firmicutes, Bacteroidetes*, *Fusobacteria*, and *Proteobacteria* constituted the majority of the microbiota on the TADs (Additional file 1: Fig. S1A). *Fusobacterium, Veillonella, Prevotella, Streptococcus, Leptotrichia,* and *Selenomonas* predominated at the genus level (Additional file 1: Fig. S1B).

**Fig. 4:**
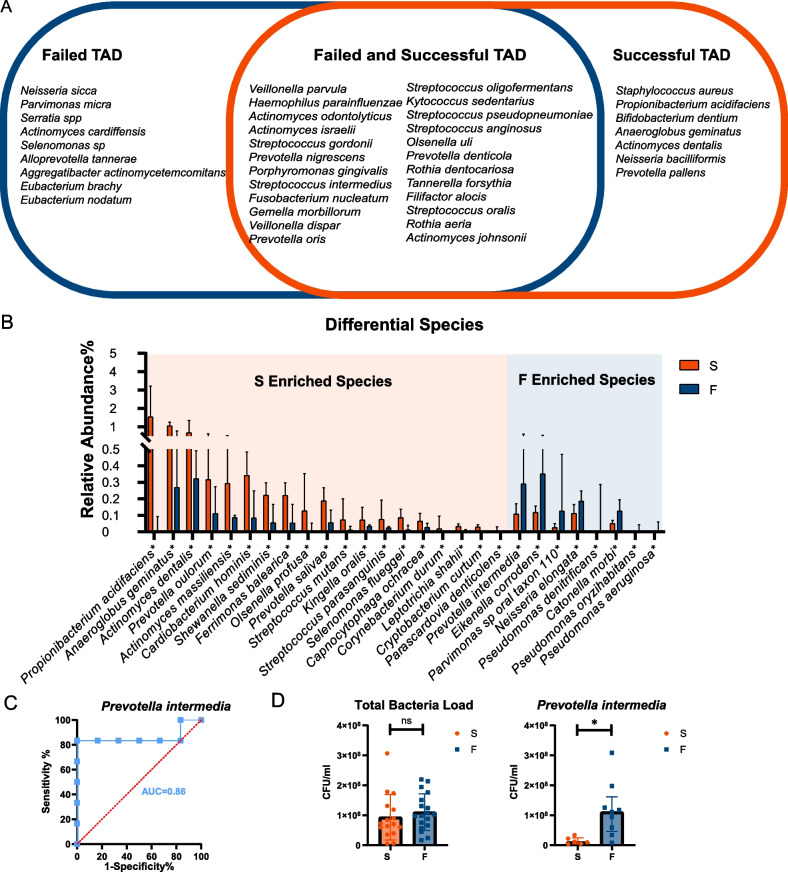
Differential species between successful and failed TADs. **A**. The most abundant species was identified through metagenomic sequencing with a relative abundance greater than 0.5%. The blue circle shows species found in failed groups. The red circle shows species found in successful groups. Species found in both groups were depicted in the mutual area. **B** Differential species with average relative abundance greater than 0.01% between successful and failed TADs based on Wilcoxon's test. **C** Receiver Operating Characteristic (ROC) analysis of *Prevotella intermedia*. **D** Quantitative real-time PCR results of total bacteria load and *Prevotella intermedia*

In metagenomic sequencing, 40 bacterial species were identified with relative abundance greater than 0.5%. Twenty-four bacterial species were found in both the failed TADs and successful TADs. Nine bacterial species were only found in the failed group and 7 bacterial species were only found in the successful group, with a relative abundance greater than 0.5% (Fig. 4A). The most abundant species included *Veillonella parvula, Haemophilus parainfluenzae, Actinomyces odontolyticus, Actinomyces israelii,* and *Streptococcus gordonii*.

### The successful and failed TAD-associated microbiota

We next compared the taxonomic composition of the microbiomes between the successful and failed TADs. We used Wilcoxon’s test to investigate the different species between failed and successful TADs. Species with a median relative abundance below 0.01% were excluded. We found that in the failed group, *Prevotella intermedia, Eikenella corrodens, Parvimonas spp, Neisseria elongata,* and *Catonella morbi* were enriched. In the successful group*, Propionibacterium acidifaciens, Anaeroglobus geminatus, Actinomyces dentalis, Prevotella oulorum, Actinomyces massiliensis, Cardiobacterium hominis, Shewanella sediminis, Ferrimonas balearica*, *Olsenella Profusa*, and *Prevotella salivae* were enriched (*p* < *0.05*). The difference demonstrated compositional changes in failed TADs (Fig. 4B).

In order to distinguish the failed and successful TADs, we conducted a Receiver Operating Characteristic (ROC) analysis (Fig. 4C) with the relative abundance of *Prevotella intermedia*. The model achieved an optimal area under the curve (AUC) value of 0.861 *(p* = *0.0374)*. The result shows the high abundance of *Prevotella intermedia* would be more likely to be found on failed TADs.

Defining the microbiome enriched in the failed group might help develop targeted anti-microbiome coating materials on TADs or give us an early screening for individuals whose TADs to be implanted are likely to fail. Aiming to verify whether *Prevotella intermedia* is enriched in the failed group, we conducted qRT-PCR in another sample group. The overall bacteria load showed no difference between successful and failed TADs (*p* = *0.251*) (Fig. 4D). In *Prevotella intermedia*, the detection rate was 50% in the failed group and 37.5% in the successful group (*p* = *0.510*). The absolute quantification of *Prevotella intermedia* was higher in detected samples in the failed group (*p* = *0.0048*) (Fig. 4D).

### The bacterial correlation analysis between successful and failed TADs

**Fig. 5:**
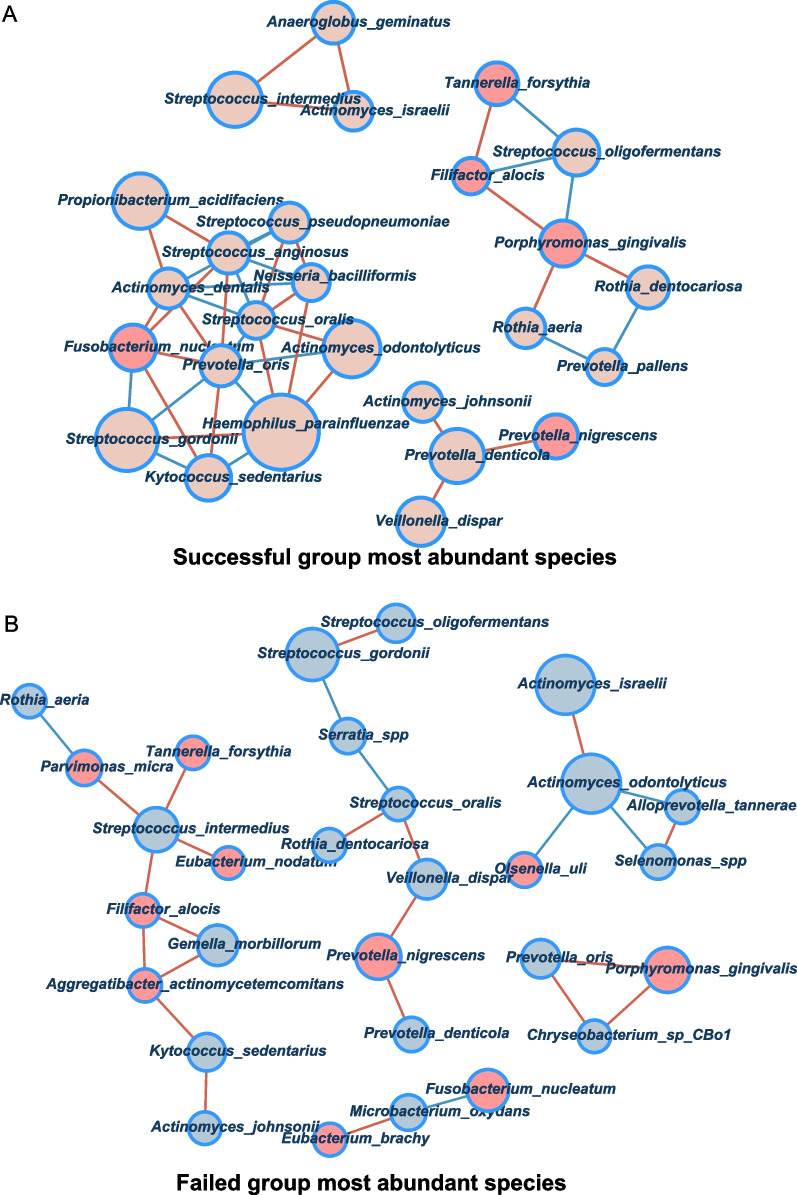
Correlations between the microbiomes on TADs. Spearman correlation coefficients > 0.4 and P-values < 0.05 are shown in the network. Red nodes represent periodontal pathogens. Lines in red between the nodes show positive correlations. Lines in blue show negative correlations. The node size is proportional to the mean abundance in the respective population

In the exploration of the relationships between microbiota species, we constructed a correlation network based on relative abundance (Fig. 5). We calculated Spearman correlations between species with relative abundance greater than 0.5%. Spearman correlation coefficients > 0.4 and P-values < 0.05 are shown in the network. From the correlation network, successful TADs demonstrated more complex relationships. On the contrary, failed TADs demonstrated more concise relationships. Notably, TADs in the failed group had stronger correlations with periodontal disease-associated taxa, such as *Fusobacterium nucleatum, Filifactor alocis, Porphyromonas gingivalis,* and *Prevotella nigrescenis*.

### Gene function was correlated with microbiota analysis on successful and failed TADs

**Fig. 6:**
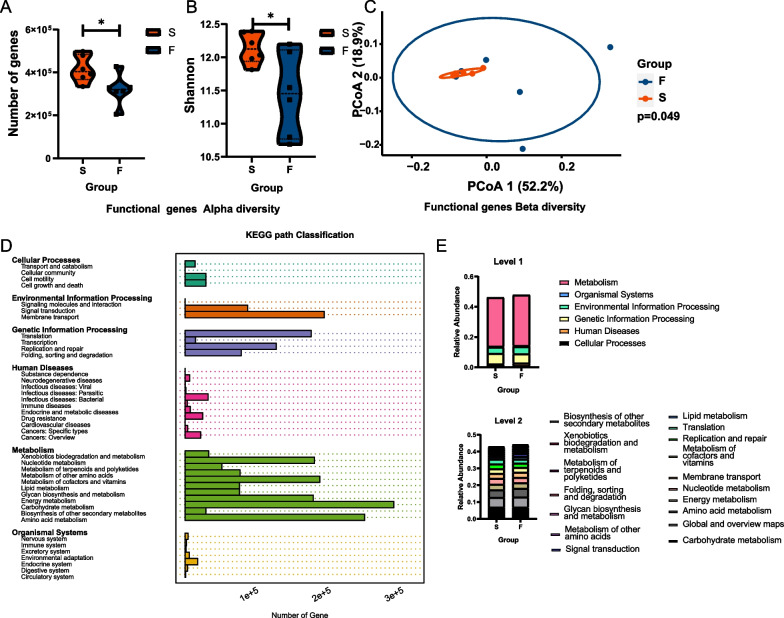
Analysis of functional genes**. A** A boxplot of the numbers of genes identified between groups (*p* = *0.031*). **B** A boxplot of α-diversity Shannon index of genes between groups (*p* = *0.030*). **C** PCoA based on functional gene distances is shown for the failed groups (blue) and successful group (red) (*p* = *0.049*). **D** KEGG classifications of functional genes. **E** KEGG classifications of functional genes categorized into level 1 and level2

We next compared the functional potentials of the microbiomes found on TADs. The functional pathways of the bacterial communities on TADs were also clearly distinctive for α-diversity indices (Gene numbers and Shannon) (*p* = *0.031, p* = *0.030*) (Fig. 6A, B) and β-diversity index (*p* = *0.049*) (Fig. 6C). KEGG (Kyoto Encyclopedia of Genes and Genomes) pathway compositions were characterized on level 1 and level 2. Both groups were enriched in carbohydrate metabolism, global and overview maps, amino acid metabolism, energy metabolism, nucleotide metabolism, membrane transport, metabolism of cofactors and vitamins, replication and repair, translation, and lipid metabolism on level 2 (Fig. 6D, E).

**Fig. 7:**
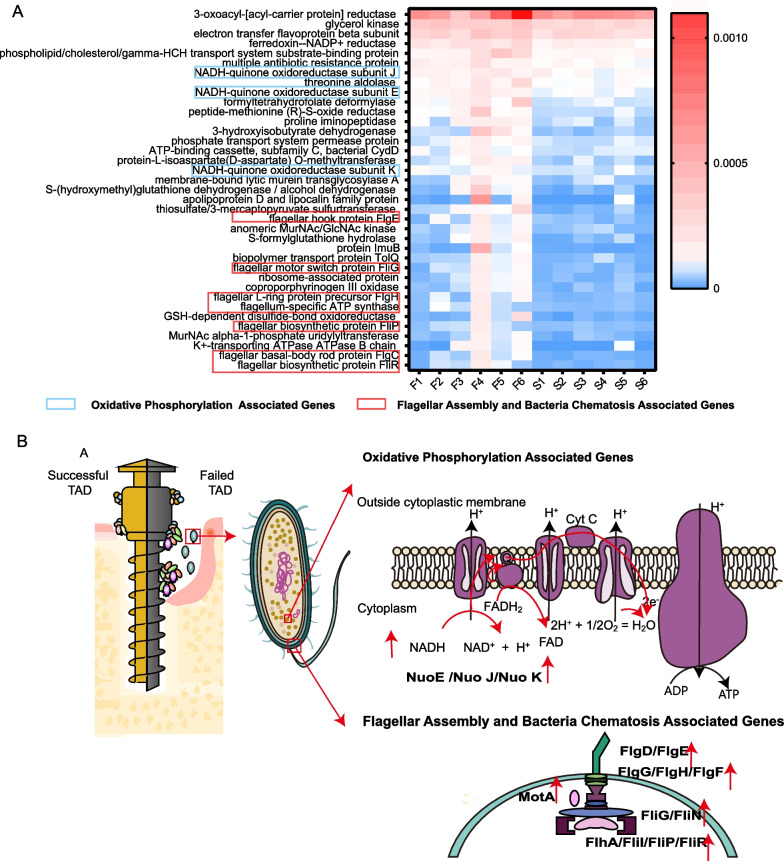
Differential functions based on the KEGG database**. A** Enriched KO pathways in failed groups. KO pathways are depicted in rows. The abundance is shown by the color gradient (blue, not detected; red, most abundant). The sample name is displayed in columns. The blue square circle KO pathways are associated with oxidative phosphorylation. The red square circle KO pathways are associated with flagellar assembly and bacterial chemotaxis. **B** Dynamics of the microbiome associated with successful and failed TADs. On successful TADs, biofilm exists on the head and the neck of the TAD. The surrounding tissue shows minor signs of inflammation. On failed TADs, biofilm also exists on the body of TAD. Peri-implant inflammation and bone resorption occur on the surrounding tissue. Failed TAD demonstrates enriched functions associated with flagellar assembly, bacterial chemotaxis, and oxidative phosphorylation. The pattern was drafted with reference to KEGG imagery[40, 41]

We next investigated enriched microbial functions of failed and successful TADs. The heatmap demonstrated the enriched functions of failed TADs. Failed TADs showed enriched KEGG pathways associated with bacterial motility. The flagellar assembly and bacterial chemotaxis-associated genes, including flagellar hook protein FlgE, flagellar motor switch protein FliG, flagellar L-ring protein precursor FlgH, flagellar biosynthetic protein FliP, flagellar basal-body rod protein FlgC, and flagellar biosynthetic protein FliR, were considered pathogenic (Fig. 7A). Failed TADs also showed enriched KEGG pathways associated with oxidative phosphorylation. This mainly included NADH-quinone oxidoreductase subunit J, NADH-quinone oxidoreductase subunit E, and NADH-quinone oxidoreductase subunit K.

## Discussion

TADs play an indispensable role in daily orthodontic practice. This study demonstrated compositional, phylogenetic, and functional differences in the microbiomes found on the surface of failed and successful TADs using 16S rRNA sequencing and metagenomic sequencing. A clear separation was seen in the clustering of these two groups, indicating differences in microbiome structure. *Prevotella intermedia, Eikenella corrodens, Parvimonas spp., Neisseria elongata,* and *Catonella morbi* were enriched in the failed group. In the correlation analysis, failed TADs demonstrated more simple inter-species relationships compared with the successful group. Considering functional aspects, the failed group showed enriched pathogenic genes involved in oxidative phosphorylation, flagellar assembly, and bacterial chemotaxis. From this analysis, we demonstrated that bacterial and functional dysbiosis occurred on failed TADs.

TADs were inserted trans-gingivally in the oral cavity and were easily contaminated by oral microbiota. Marked distinctions were observed for species associated with periodontitis and peri-implantitis compared to healthy individuals based on microbiome analysis [25]. Peri-implantitis is a polymicrobial disease caused by plaque accumulation and retention. The ecological imbalance around implants has been shown to be related to abnormal changes in bacterial correlation, community structure, and local stability [26, 27]. As TADs and dental implants are all titanium, we hypothesized that microbiome contamination around failed TADs might occur. However, it is important to note that TADs and dental implants are different in the insertion site. There is also no osseointegration in the TAD insertion site. Therefore, microorganisms inducing TAD failure might not be analogous to peri-implantitis. In our study, *Prevotella intermedia, Eikenella corrodens, Neisseria elongata,* and *Catonella morbi* were enriched around failed TADs. These bacteria were gram-negative species that could induce host inflammation.

We observed different microbial compositions around successful and failed TADs based on NGS technology. The results of our study did not agree with the previous studies [12–14]. Other studies tried to explain TAD failure from the perspective of well-known periodontal pathogens. Tortamano et al*.* (2012) and Apel et al*.* (2009) implemented polymerase chain reaction (PCR) to analyze failed TADs and successful TADs [12, 13], but no statistical difference was found in the detection rate of *Porphyromonas gingivalis* and *Aggregatibacter actinomycetemcomitans*, which are core members of the microbiome in periodontitis and peri-implantitis disease. Andrucioli et al. (2018) implemented a checkerboard DNA-DNA hybridization technique to detect the 40 bacterial species classified by microbial complexes proposed by Socransky et al. [28] in periodontitis. These complexes included the *actinomyces* group, purple, yellow, green, orange, and red complexes, and other species. No significant difference was found with respect to microbial complexes [14].

The reason for this disagreement may be due to the implementation of PCR and checkerboard DNA-DNA hybridization that could only detect well-known species in the oral cavity. Moreover, the methods used in previous research were not quantitative analyses. The implementation of next-generation sequencing, however, allowed us to address the question from a macro perspective. We discovered quantitative distinctions between the microbiome structure and composition of failed and successful TADS. The application of NGS could also detect bacteria that were not extensively studied before. The application of NGS allowed us to discover *Eikenella corrodens, Neisseria elongata, Catonella morbi* in the oral cavity. Our study utilized a significant sample size, with 135 TADs incorporated for metagenomic sequencing. This sample size allowed for the amplification of small changes in the microbiome communities. Microbiome sampling in some of the previous studies relied on paper cones to collect samples from the peri-implant sulcus. In our study, TADs were removed and immediately immersed in a normal saline solution. This sampling method optimized the number of bacteria detected.

In addition, bacterial secretion of molecules, such as lipopolysaccharides, lipoproteins, and lipopeptides, might contribute to the host inflammatory response and induce the production of osteolytic agents that enhance bone resorption [29]. These components could produce an arsenal of virulence factors, such as lipopolysaccharides, lectins, and cilia, that could directly destroy periodontal tissue or stimulate the host immune-inflammatory response and lead to bone destruction [30]. Studies have highlighted the strong role of *Prevotella intermedia* in peri-implantitis [31, 32]. *Prevotella intermedia* was present in low abundance in TAD samples. However, pathogenic bacteria do not need large biomass to produce marked effects. In the case of periodontitis, a low abundance of pathogens might ensure higher community productivity compared to dominant community members [23].

Virulence related functional modules, namely flagellar systems and bacterial chemotaxis, are important for bacterial colonization and infection. These functions were shown to be upregulated in oral diseases such as periodontitis [33]. In our analysis, genes related to these functions were enriched in failed TADs. Bacterial swimming and swarming are necessary for bacteria to live in various environments and play multiple roles in pathogenesis, including reaching optimal host location and invasion [34]. There is a growing number of evidence that suggest flagella motility, and chemotaxis are all critical to the formation of biofilms in various stages [35]. The enrichment of these functions on TAD plaque could potentially increase plaque formation and induce instability of TADs. In addition, we also detected enriched genes for oxidative phosphorylation. NADH dehydrogenase is the largest complex in the electron transport chain and is very important for energy generation [36]. This study found that NADH dehydrogenase-related genes in the electron transport chain of the tricarboxylic acid cycle were significantly increased in the biofilms on failed TADs. Therefore, we speculate that energy metabolism is more active in the failed TAD group. The mechanism of how these pathways influenced the stability of TADs is characterized in Fig. 7B.

In this analysis, we sought to relate the failure of TADs with differences in the oral cavity microbiota communities. The failure of TADs has multifactorial etiologies, in which inflammation has been proven to be an important cause. The elevated function of TAD microorganisms could possibly trigger an immune response from the host. Previous studies have detected the expression of ILs, TNF-α, RANKL, MMP-2, and MMP-9 via JNK, Erk1/2, Wnt5a, NF-κBp65, OPN, and TAB/TAK signaling pathways and suggested IL-1β and IL-6 be the critical inflammation factors inducing the inflammatory reaction surrounding implants [11]. Other studies have focused on the host MicroRNA expression and proposed miR-4291, miR-1245b-3p, and miR-1825 as potential diagnostic markers and potential therapeutic targets for inflammation around TAD [37]. The dynamics of TAD failure might not be similar to that of periodontitis and peri-implantitis, as periodontitis and peri-implantitis generally take years to develop while TADs only function during orthodontic treatment, normally in a year or two. It has been suggested that inflammation around TADs may not be primarily caused by bacterial infection. On the other hand, it may primarily be caused by foreign body implantation. The bacterial infection could hamper the healing process and provide a poor body-seal environment on the neck of the TADs, further allowing for more bacterial invasion and triggering host inflammatory responses [11, 38]. Our study showed that the dysbiosis of oral microbiota in failed TADs was not identical in each case. The microflora of successful TADs was similar and stable, as inter-group discrepancies were small and the predominant species showed strong correlations. In failed TADs, the variation within the group was significant and the correlations were weak. This result indicates that a variety of pathogens could all contribute to the failure of TADs. Therefore, it is critical to comprehend the microbiological factors underlying TAD failure and develop methods to reduce bacterial infection.

This study also had limitations. First, considering the difficulties in acquiring the samples, the verification sample size was rather limited. A larger population is in need to testify to the potentially pathogenic bacteria presented in this analysis. Second, this analysis did not establish a link between microbiome infection, inflammation, and TAD stability. Exploration into the mechanism behind the pathogenic effect of the failure-associated species and pathways will improve our knowledge of how microbiota communities influence TAD failure. This may lead to the development of novel antibacterial materials and reduce the failure rate of TADs. Additionally, it is very difficult to use the traditional sampling method via a paper point for the sampling of microbiomes around TAD due to the constraint of TAD size and the amount of DNA requested for sequencing. Finally, just as studies in peri-implantitis [39], further investigation of the relationship between the host immune molecules and specific taxa will provide insights into the interactions between the oral microbiome and the host immune system.

## Conclusions

In conclusion, this analysis elucidated the overall compositional and functional differences of bacteria on failed TADs and successful TADs by next-generation sequencing. This study highlights the importance of controlling bacterial adhesion on TAD surfaces.

## Supplementary Information

**Additional file 1:** The most abundant genera and phyla in 16S rRNA gene sequencing.

## Data Availability

The datasets generated and/or analyzed during the current study are available in the NCBI repository under PRJNA851548 (https://www.ncbi.nlm.nih.gov/bioproject/PRJNA851548).

## Abbreviations

TADs: Temporary anchorage devices
qRT-PCR: Quantitative real-time polymerase chain reaction
NGS: Next-generation sequencing

## References

[1] Antoszewska-Smith J, Sarul M, Lyczek J, Konopka T, Kawala B (2017). Effectiveness of orthodontic miniscrew implants in anchorage reinforcement during en-masse retraction: a systematic review and meta-analysis. Am J Orthodont Dentofac Orthoped.

[2] Alharbi F, Almuzian M, Bearn D (2019). Anchorage effectiveness of orthodontic miniscrews compared to headgear and transpalatal arches: a systematic review and meta-analysis. Acta Odontol Scand.

[3] Reynders R, Ronchi L, Bipat S (2009). Mini-implants in orthodontics: a systematic review of the literature. Am J Orthodont Dentofac.

[4] Aly SA, Alyan D, Fayed MS, Alhammadi MS, Mostafa YA (2018). Success rates and factors associated with failure of temporary anchorage devices: a prospective clinical trial. J Investig Clin Dent.

[5] Papadopoulos MA, Papageorgiou SN, Zogakis IP (2011). Clinical effectiveness of orthodontic miniscrew implants: a meta-analysis. J Dent Res.

[6] Chen YJ, Chang HH, Lin HY, Lai EH, Hung HC, Yao CC (2008). Stability of miniplates and miniscrews used for orthodontic anchorage: experience with 492 temporary anchorage devices. Clin Oral Implant Res.

[7] Lai T-T, Chen M-H (2014). Factors affecting the clinical success of orthodontic anchorage: experience with 266 temporary anchorage devices. J Dent Sci.

[8] Papageorgiou SN, Zogakis IP, Papadopoulos MA (2012). Failure rates and associated risk factors of orthodontic miniscrew implants: a meta-analysis. Am J Orthodont Dentofac Orthoped.

[9] Hong SB, Kusnoto B, Kim EJ, BeGole EA, Hwang HS, Lim HJ (2016). Prognostic factors associated with the success rates of posterior orthodontic miniscrew implants: a subgroup meta-analysis. Korean J Orthodont.

[10] Rosier BT, Marsh PD, Mira A (2018). Resilience of the oral microbiota in health: mechanisms that prevent dysbiosis. J Dent Res.

[11] He W, Zhu H, Liu C (2021). Profiles of inflammation factors and inflammatory pathways around the peri-miniscrew implant. Histol Histopathol.

[12] Apel S, Apel C, Morea C, Tortamano A, Dominguez GC, Conrads G (2009). Microflora associated with successful and failed orthodontic mini-implants. Clin Oral Implant Res.

[13] Tortamano A, Dominguez GC, Haddad AC, Nunes FD, Nacao M, Morea C (2012). Periodontopathogens around the surface of mini-implants removed from orthodontic patients. Angle Orthod.

[14] Andrucioli MCD, Matsumoto MAN, Saraiva MCP, Feres M, Figueiredo LCd, Sorgi CA, Faccioli LH, Silva RABd, Silva LABd, Nelson-Filho P: Successful and failed mini-implants: microbiological evaluation and quantification of bacterial endotoxin. *J Appl Oral Sci* 2018, 26(0). 10.1590/1678-7757-2017-0631 PMC6025887 29995147

[15] Kirst ME, Li EC, Alfant B, Chi YY, Walker C, Magnusson I, Wang GP (2015). Dysbiosis and alterations in predicted functions of the subgingival microbiome in chronic periodontitis. Appl Environ Microbiol.

[16] Genco RJ, LaMonte MJ, McSkimming DI, Buck MJ, Li L, Hovey KM, Andrews CA, Sun Y, Tsompana M, Zheng W (2019). The subgingival microbiome relationship to periodontal disease in older women. J Dent Res.

[17] Zheng H, Xu L, Wang Z, Li L, Zhang J, Zhang Q, Chen T, Lin J, Chen F (2015). Subgingival microbiome in patients with healthy and ailing dental implants. Sci Rep.

[18] Wang Q, Garrity GM, Tiedje JM, Cole JR (2007). Naive Bayesian classifier for rapid assignment of rRNA sequences into the new bacterial taxonomy. Appl Environ Microbiol.

[19] Dewhirst FE, Chen T, Izard J, Paster BJ, Tanner AC, Yu WH, Lakshmanan A, Wade WG (2010). The human oral microbiome. J Bacteriol.

[20] Liu YX, Qin Y, Chen T, Lu M, Qian X, Guo X, Bai Y: A practical guide to amplicon and metagenomic analysis of microbiome data. Protein Cell 2020:1–16. 10.1007/s13238-020-00724-8 PMC8106563 32394199

[21] Qin N, Yang F, Li A, Prifti E, Chen Y, Shao L, Guo J, Le Chatelier E, Yao J, Wu L (2014). Alterations of the human gut microbiome in liver cirrhosis. Nature.

[22] Lukic D, Karygianni L, Flury M, Attin T, Thurnheer T: Endodontic-like oral biofilms as models for multispecies interactions in endodontic diseases. Microorganisms 2020, 8(5). 10.3390/microorganisms8050674 PMC7285038 32384777

[23] Abusleme L, Dupuy AK, Dutzan N, Silva N, Burleson JA, Strausbaugh LD, Gamonal J, Diaz PI (2013). The subgingival microbiome in health and periodontitis and its relationship with community biomass and inflammation. ISME J.

[24] Lopes MP, Cruz AA, Xavier MT, Stocker A, Carvalho-Filho P, Miranda PM, Meyer RJ, Soledade KR, Gomes-Filho IS, Trindade SC (2020). Prevotella intermedia and periodontitis are associated with severe asthma. J Periodontol.

[25] Ghensi P, Manghi P, Zolfo M, Armanini F, Pasolli E, Bolzan M, Bertelle A, Dell'Acqua F, Dellasega E, Waldner R (2020). Strong oral plaque microbiome signatures for dental implant diseases identified by strain-resolution metagenomics. NPJ Biofilms Microbiomes.

[26] Zhang Y, Li Y, Yang Y, Wang Y, Cao X, Jin Y, Xu Y, Li SC, Zhou Q (2021). Periodontal and peri-implant microbiome dysbiosis is associated with alterations in the microbial community structure and local stability. Front Microbiol.

[27] Belibasakis GN, Manoil D (2021). Microbial community-driven etiopathogenesis of peri-implantitis. J Dent Res.

[28] Socransky SS, Haffajee AD, Cugini MA, Smith C, Kent RL (1998). Microbial complexes in subgingival plaque. J Clin Periodontol.

[29] Henderson B, Kaiser F (2018). Bacterial modulators of bone remodeling in the periodontal pocket. Periodontol.

[30] Lafaurie GI, Sabogal MA, Castillo DM, Rincon MV, Gomez LA, Lesmes YA, Chambrone L (2017). Microbiome and microbial biofilm profiles of peri-implantitis: a systematic review. J Periodontol.

[31] de Waal YC, Eijsbouts HV, Winkel EG, van Winkelhoff AJ (2017). Microbial characteristics of peri-implantitis: a case-control study. J Periodontol.

[32] Sahrmann P, Gilli F, Wiedemeier DB, Attin T, Schmidlin PR, Karygianni L: The microbiome of peri-implantitis: a systematic review and meta-analysis. Microorganisms 2020, 8(5). 10.3390/microorganisms8050661 PMC7284896 32369987

[33] Deng ZL, Szafranski SP, Jarek M, Bhuju S, Wagner-Dobler I (2017). Dysbiosis in chronic periodontitis: Key microbial players and interactions with the human host. Sci Rep.

[34] Morimoto YV, Minamino T, Harris JR, Marles-Wright J (2021). Architecture and assembly of the bacterial flagellar motor complex. Macromolecular protein complexes III: structure and function.

[35] Colin R, Ni B, Laganenka L, Sourjik V: Multiple functions of flagellar motility and chemotaxis in bacterial physiology. FEMS Microbiol Rev 2021, 45(6). 10.1093/femsre/fuab038 PMC8632791 34227665

[36] Ito T, Gallegos R, Matano LM, Butler NL, Hantman N, Kaili M, Coyne MJ, Comstock LE, Malamy MH, Barquera B: Genetic and biochemical analysis of anaerobic respiration in bacteroides fragilis and its importance in vivo. mBio 2020, 11(1). 10.1128/mBio.03238-19 PMC7002350 32019804

[37] He W, Yang Y, Cai L, Lei Q, Wang Z, Che X (2021). MicroRNA expression profiles in peri-miniscrew implant crevicular fluid in orthodontics: a pilot study. BMC Oral Health.

[38] Andrucioli MCD, Matsumoto MAN, Fukada SY, Saraiva MCP, Bergamo AZN, Romano FL, Silva R, Silva L, Nelson-Filho P (2019). Quantification of pro-inflammatory cytokines and osteoclastogenesis markers in successful and failed orthodontic mini-implants. J Appl Oral Sci.

[39] Schminke B, Vom Orde F, Gruber R, Schliephake H, Burgers R, Miosge N (2015). The pathology of bone tissue during peri-implantitis. J Dent Res.

[40] Kanehisa M, Goto S (2000). KEGG: kyoto encyclopedia of genes and genomes. Nucleic Acids Res.

[41] Kanehisa M (2019). Toward understanding the origin and evolution of cellular organisms. Protein Sci.

