# Supplementary material for: Oral microbiome contributes to the failure of orthodontic temporary anchorage devices (TADs)
Source: BMC Oral Health. 2023 Jan 17;23:22. doi: 10.1186/s12903-023-02715-7 (PMC9844000; doi:10.1186/s12903-023-02715-7)
Supplement: Supplementary file 1 — Additional file 1: The most abundant genera and phyla in 16S rRNA gene sequencing. [file 12903_2023_2715_MOESM1_ESM.pdf]

Appendix Figure 1

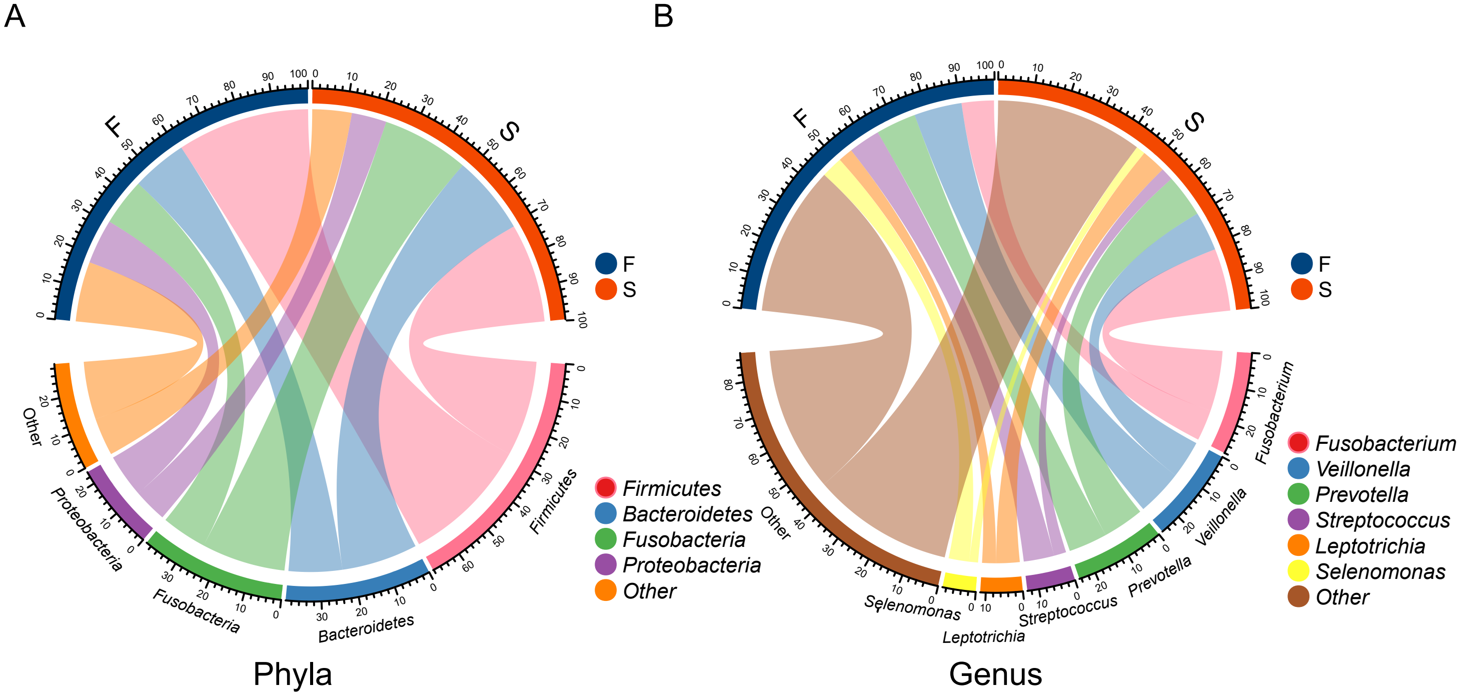

Appendix Figure 1 The most abundant genera and phyla based on 16S rRNA gene sequencing.
